# Health-promoting factors in medical students and students of science, technology, engineering, and mathematics: design and baseline results of a comparative longitudinal study

**DOI:** 10.1186/1472-6920-14-134

**Published:** 2014-07-04

**Authors:** Thomas Kötter, Yannick Tautphäus, Martin Scherer, Edgar Voltmer

**Affiliations:** 1 Institute for Social Medicine and Epidemiology, University Medical Center Schleswig-Holstein, Campus Lübeck, Ratzeburger Allee 160, Lübeck 23562, Germany; 2 Department of Primary Medical Care, University Medical Center Hamburg-Eppendorf, Martinistraße 52, Hamburg 20246, Germany; 3 Department of Health and Behavioural Sciences, Friedensau Adventist University, An der Ihle 19, Friedensau 39291, Germany

**Keywords:** Medical students, Medical education, Mental health, Health promotion, Prevention, Personality assessment

## Abstract

**Background:**

The negative impact of medical school on students' general and mental health has often been reported. Compared to students of other subjects, or employed peers, medical students face an increased risk of developing depression, anxiety and burnout. While pathogenetic factors have been studied extensively, less is known about health-promoting factors for medical students' health. This longitudinal study aims to identify predictors for maintaining good general and mental health during medical education. We report here the design of the study and its baseline results.

**Methods:**

We initiated a prospective longitudinal cohort study at the University of Lübeck, Germany. Two consecutive classes of students, entering the university in 2011 and 2012, were recruited. Participants will be assessed annually for the duration of their course. We use validated psychometric instruments covering health outcomes (general and mental health) and personality traits, as well as self-developed, pre-tested items covering leisure activities and sociodemographic data.

**Results:**

At baseline, compared to students of STEM (science, technology, engineering, and mathematics) subjects (n = 531; 60.8% response rate), a larger proportion of medical students (n = 350; 93.0% response rate) showed good general health (90.9% vs. 79.7%) and a similar proportion was in good mental health (88.3% vs. 86.3%). Medical students scored significantly higher in the personality traits of extraversion, conscientiousness, openness to experience and agreeableness. Neuroticism proved to be a statistically significant negative predictor for mental health in the logistic regression analyses. Satisfaction with life as a dimension of study-related behaviour and experience predicted general health at baseline. Physical activity was a statistically significant predictor for general health in medical students.

**Conclusions:**

Baseline data revealed that medical students reported better general and similar mental health compared to STEM students. The annual follow-up questionnaires, combined with qualitative approaches, should clarify wether these differences reflect a higher resilience, a tendency to neglect personal health problems - as has been described for physicians - before entering medical school, or both. The final results may aid decision-makers in developing health-promotion programmes for medical students.

## Background

A growing body of evidence shows that medical school can pose a threat to students’ mental health. Longitudinal studies in different countries revealed that the mental health of medical students declines during their graduate education, while the prevalence of depression, anxiety or burnout increases [1-6]. Numerous studies have examined potential pathogenetic factors for students’ mental health [5,7,8]. Workload, competition, sleep deprivation, lack of social support and the suffering and dying of patients contribute to medical students’ stress and their deteriorating mental health [8]. Other predictors for graduating from medical school in poor mental health have been shown to include certain personality traits [5] and coping styles [9]. Students with high scores in neuroticism, overcommitment or emotional-oriented coping may be at a higher risk for psychosocial symptoms and illness. In their systematic review on the influence of personality characteristics on medical students’ performance, Doherty and Nugent found conscientiousness to be a significant predictor not only of academic success in medical school, but also of vulnerability to stress [10]. Certain pathogenetic factors specific to medical education seem to lead to a higher prevalence of stress-related illnesses, such as depression, anxiety and burnout among medical students when compared to students of other subjects or employed peers [8]. However, a recent systematic review conducted by Cohen et al. [11] questions wether the prevalence of these conditions is higher for medical students than for the general population. Nevertheless, being already burned out or depressed at the beginning of a medical career could have negative consequences not only for the impaired doctor him- or herself, but also for their patients. Doctors’ distress may lead to decreased patient satisfaction [12] and decreased patient compliance [13], as well as increased absenteeism and cynicism among doctors. Even more importantly, recent studies have shown that physicians’ distress is related to medical errors [14,15].

One promising strategy in combating the decrease in mental health during medical school might be to promote good health. Focusing on resources which might be effective in promoting resilience, rather than on risk factos for illness, is a widely accepted public health concept [16]. Health-promoting factors can include - amongst others - optimism, hardiness, self-efficacy, a sense of coherence and social support [1]. Since the medical knowledge that is required to be learned is not arbitrarily reducible, and since it lies in the nature of medicine to act with the highest responsibility, to often deal with uncertainty, and to be confronted with the suffering and dying of patients, fostering resilience could thus be a promising approach to solving the particular problem of medical students’ deteriorating mental health. However, this certainly does not exclude fostering health by setting-related interventions targeted to focus on the curriculum, or fostering team orientation instead of competition, for example through mentoring programs and by critically reviewing the frequency and content of examinations.

Mental health is an integral part of health as defined by the World Health Organization (WHO) [17]. Good mental health is the foundation of good “general” health and both are interdependent. However, to date, little attention has been given to the general health of medical students and its changes during medical education. We are not aware of any longitudinal studies reporting on the general health of medical students as an outcome. Also, to date, little is known about protective factors for medical students’ health [3,7]. In addition to a few cross-sectional studies [18,19], we are aware of only one longitudinal study reporting predictors for resilience in medical students [3]. Since promoting health and preventing health deterioration is the preferable action strategy compared to treating already-ill students or doctors, more research in this field is overdue.

Our study, therefore, has three major aims:

1) to identify individual predictors of graduating from medical school in good general health (defined as rating one’s health [in general] as good or very good) and good mental health (defined as the absence of psychological distress [anxiety and/or depression] [20]);

2) to reveal starting points for health-promoting interventions in medical school;

3) to evaluate the feasibility and effectiveness of pilot interventions initiated during the study period.

Here we present the protocol and baseline data of our longitudinal study.

## Methods

### Study design and setting

#### *Study design*

We are conducting a single centre, prospective, observational study. From their enrolment onwards, students will be surveyed annually for the duration of their undergraduate and graduate education.

#### *Setting*

The study center is the University of Lübeck, a public university with a focus on medicine and life sciences.

### Participants

We invited all Freshmen from the 2011 and 2012 cohorts (two consecutive classes) at the University of Lübeck by advertising during the central inauguration ceremony. The baseline surveys were taken in October 2011 and October 2012 during the pre-course week. There were no exclusion criteria.

#### *Study size*

The study size is predefined by the number of Freshmen at the University of Lübeck. In order to achive a good compromise between homogeneity and study size, we recruited two complete, consecutive classes.

#### *Bias*

In order to reduce bias due to non-response, we offer a reward in terms of a book voucher for the amount of 5 Euro per survey for every participant. Information on the whereabouts of non-responders in the follow-up survey will be provided by the university administration in order to interpret losses to follow-up. Additionally, we offer a short non-responder survey for students unwilling to participate in the baseline survey.

### Data collection/instruments

The questionnaires comprise sociodemographic questions, health outcomes (self-rated general health and mental health as defined above) as well as potential predictors of the outcomes (dimensions of study-related behaviour and experience, personality traits and self-developed, pre-tested items covering leisure activity). The pre-tested, paper-based baseline surveys consisted of 150 items and took about 30 minutes to complete. All follow-up surveys will be websurveys and are planned to be shorter.

#### *Sociodemographic data*

Sociodemographic data comprises age, gender, marital status, number of children, and distance to the parental home (calculated using the postal code).

#### *General health*

In order to measure self-rated general health by a single item, as suggested by the World Health Organisation (WHO; [21]) and previous research [22], we use the question: “How would you describe your health in general?”, with the five answering options: “very good” (1), “good”, “fair”, “poor”, and “very poor” (5) [21]. In the analyses reported here, being healthy in general was defined as rating one’s general health as “good” or “very good”.

#### *Mental health*

We used the Hospital Anxiety and Depression Scale (HADS) in order to measure mental health, which we defined as the absence of psychological distress, i.e. the absence of both clinical anxiety and depression [20]. The HADS was initially developed for clinical populations but has been widely used among students in general and medical students in particular [23-27]. The HADS comprises fourteen items for two sub-scales. Each of the two sub-scales relates to anxiety and depression and consists of seven items which obtain responses on a four-point Likert scale, ranging from 0 = mostly to 3 = not at all. Possible sub-scale scores range from 0 to 21. We used the German version (HADS-D), published by Herrmann-Lingen et al. in 1995 and available in the 3^rd^ edition [28]. For statistical purposes, HADS scores were, and will be, dichotomized (anxiety or depression negative). Students with a depression score >8 were considered depressive, and students with an anxiety score of >10 were considered anxious [28]. In the analyses reported here, being mentally healthy was defined as being neither depressive nor anxious.

#### *Study-related behaviour and experience patterns*

The AVEM (“Arbeitsbezogene Verhaltens- und Erlebensmuster”, [Work-related Behaviour and Experience Patterns] [29]) was originally developed to collect self-reported data about personal experiences with work-related stress and typical coping strategies. We used the 44-item version adapted for students (see Additional file 1) [1]. This instrument comprises 11 separate dimensions representing behaviour-related health risks or resources (see Table 1). These scales cover the following three major domains: (1) professional commitment; (2) resistance towards stress; and (3) emotional well-being (in the context of work). Each scale comprises 4 items with response options presented as 5-point Likert scales, ranging from 1: I strongly disagree to 5: I strongly agreee. The AVEM has been used with medical students in numerous studies conducted in Germany [1,30,31].

**Table 1:** Characteristics of the study participants at baseline

	**Medical students**			**STEM students**			**Overall**
	**2011 (n = 178)**	**2012 (n = 172)**	**Overall (n = 350)**	**2011 (n = 298)**	**2012 (n = 233)**	**Overall (n = 531)**	**(n = 881)**
Response (%)	93.2	93.0	93.1	77.9	54.0	60.8	70.5
Mean age in years (SD)	21.0 (3.4)	20.8 (3.0)	20.9 (3.2)	20.3 (2.2)	20.0 (2.1)	20.1 (2.2)	20.4 (2.7)
Sex (% female)	65.2	67.4	66.3	41.3	52.4	46.1	54.2
Distance to parental home (km; median, range)	196, 0-709	234, 0-687	230, 0-709	71, 0-698	138, 0-716	94, 0-716	144, 0-716
Sports (1 hr or more/week, %)	78.1	78.4	78.2	65.9	64.8	65.4	70.5
Musical activity (any, %)	57.3	62.2	59.7	48.8	48.7	48.8	53.1
Activity in a religious community (1 hr or more/week, %)	9.0	8.7	8.9	4.4	4.3	4.4	6.2
Relaxation technique (1 hr or more/week, %)	5.6	7.0	6.3	10.1	4.8	7.8	7.2
Voluntary services (1 hr or more/week, %)	21.9	28.7	25.2	17.5	12.6	15.4	19.3

#### *Personality traits*

Personality traits predict health outcomes [32]. The NEO FFI is a short version (60 items) of the NEO PI-R (240 items) and measures the Big Five domains of personality (neuroticism, extraversion, openness to experience, conscientiousness and agreeableness) [33]. Each of the five sub-scales consists of 12 items that obtain responses on a five-point Likert scale, ranging from 0 = I strongly disagree to 5 = I strongly agree. The two questionnaires have been used with medical students in numerous studies [5,34-36].

#### *Leisure activity*

Furthermore, we assess certain leisure activities, which could serve as protective factors for the students’ self-rated general and mental health [3]. In the baseline survey, we asked whether students were involved in sports, musical activities, voluntary services, activity in religious communities and about the practice of relaxation techniques, such as yoga or progressive muscle relaxation. Each of these activities has been proposed as a health-promoting factor among students and the general population [37-43]. Leisure activity will be assessed every other year in the follow-up surveys.

### Data management

From the paper-based baseline surveys, data was input to a Microsoft Access database via an entry mask resembling the original questionnaire. Following a random quality control by a second researcher, data was exported to IBM SPSS statistics, version 20. From the websurvey, data is exported directly into an SPSS format file.

Data from the follow-up surveys will be matched by the pseudonyms explained below.

### Data analysis

After a plausibility check, data from the baseline questionnaires was merged and summarised using descriptive statistics to provide a comprehensive profile of this cohort. Where possible, we substitute missing values following the rules provided in the handbooks for the instruments (see above). We then exclude incomplete data-sets. Data analyses are conducted with IBM SPSS statistics, version 20. We use two-tailed t-tests to compare means of continuous variables and report results as means ± standard deviation. For categorical variables, data are analysed using chi-square-tests and results reported as percentages. In order to express bivariate correlations, we use Spearman’s rho. We use binary logistic regression analyses in order to assess correlations between potential predictors and outcomes. We conduct separate regression analyses for both groups of students (medical and STEM students). In order to remove variables in the logistic regression analyses, we employ stepwise backwards elimination with p > 0.05 as the level for removing effects, while keeping age and gender as important sociodemographic variables and the other outcome (general or mental health) in order to control for them. Additional statistical methods used for the longitudinal analyses will be detailed in the respective publications.

### Ethical issues

This study is being conducted in accordance with the guidelines provided by the Declaration of Helsinki. The study has been approved by the University’s Deans and President and the Student Council and we have obtained informed consent from each participant. All data are collected de-identified. The de-identification is stressed in both the advertisements for the baseline and follow-up surveys as well as for the informed consent sheet. A self-generated code and the student’s matriculation number serve as pseudonyms, both of which cannot be linked to the real name by the investigators without further investigation. The study protocol was approved by the ethical committee of the University of Lübeck (file reference: 11-010).

## Results

In the following sections, we present and discuss cross-sectional baseline results of our ongoing longitudinal study.

### Participants

In 2011 and 2012, 351 medical students and 540 STEM students were recruited to participate in our study. Data from 350 medical students and 531 STEM students were included in the statistical analyses. After exclusion of incomplete data-sets, the response rates were 93.0% for medical students and 60.8% for STEM students. The average age of the study participants at baseline was 20.43 ± 2.65 years with a range of 16 to 41. The majority of the medical students (66.3%; whole student population: 65.8%) and 46.1% of the STEM students (whole student population: 41,1%) were female. Compared to STEM students, a higher percentage of medical students exercised regularly (78.2% vs 65.4%) or engaged in musical acticity (59.7% vs 48.8%). Only a few were engaged in a religious community or practiced a relaxation technique regularly.

Further demographic data are shown in Table 1.

### Dimensions of study-related behaviour and experience

Table 2 shows the sum-scores in the dimensions of study-related behaviour and experience of the medical students compared to the STEM students at baseline.

**Table 2:** Dimensions of study-related behaviour and experience patterns at baseline

**Dimension**	**Medical students**		**STEM students**			
	**Mean**	**SD**	**Mean**	**SD**	**t-Value**	**p-value**
Subjective significance of work	12.05	3.06	11.47	3.19	-2.68	<0.01
Career ambition	15.31	2.71	14.56	3.00	-3.79	<0.01
Tendency to exert	12.45	2.98	11.23	3.33	-5.66	<0.01
Striving for perfection	14.89	2.99	13.78	3.13	-5.24	<0.01
Emotional distancing	12.95	2.56	13.52	2.75	3.14	<0.01
Resignation tendencies	11.80	3.20	11.43	3.26	-1.66	0.10
Offensive coping with problems	13.72	2.70	12.79	2.77	-4.97	<0.01
Balance and mental stability	13.34	3.09	13.63	3.27	1.34	0.18
Satisfaction with work	17.07	2.74	15.15	2.96	-9.86	<0.01
Satisfaction with life	17.36	2.62	15.94	2.70	-7.71	<0.01
Experience and social support	18.05	2.17	17.40	2.81	-3.90	<0.01

Medical students showed statistically significant higher sum-scores in the dimensions of subjective significance of work, career ambition, tendency to exert, striving for perfection, offensive coping with problems, satisfaction with work, satisfaction with life and experience of social support. STEM students, in contrast, showed statistically significant higher sum-scores in the dimension of emotional distancing. We observed no statistically significant difference between the two groups regarding the dimensions of resignation tendency and balance and mental stability.

### Personality traits

Mean values for the five personality traits are shown in Table 3. Medical students scored significantly higher than STEM students in all but one dimension of personality: extraversion, openness to experience, conscientiousness and agreeableness. The mean scores in the NEO-FFI dimension of neuroticism did not differ significantly between the two groups.

**Table 3:** Personality traits of medical and STEM students

**Trait**	**Medical students**		**STEM students**			
	**Mean**	**SD**	**Mean**	**SD**	**t-value**	**p-value**
Neuroticism	18.71	7.65	19.76	7.42	2.01	0.04
Extraversion	30.63	6.14	28.10	6.69	-5.67	<0.01
Openness to experience	32.15	6.13	30.79	6.05	-3.25	<0.01
Agreeableness	34.37	5.47	32.31	5.78	-5.28	<0.01
Conscientiousness	33.85	5.63	30.97	6.73	-6.85	<0.01

### General and mental health

Self-rated general health was slightly, but significantly, different between STEM and medical students. Medical students rated their general health better (1.73 ± 0.64) than STEM students (1.98 ± 0.76) (p < 0.01; Cohen’s d -0.36). 90.9% of the medical and 79.7% of the STEM students were in good general health (as defined above) before the beginning of the courses (p < 0.01).

When compared to STEM students, a significantly higher proportion of medical students was free of clinically-relevant depression (as defined above) (96.6% vs 92.2%; p = 0.02). The proportions for absence of clinically-relevant anxiety (as defined above) didn’t differ significantly between medical and STEM students (89.7% vs 90.0%; p = 0.91).

A higher proportion of the medical students (88.3% vs 86.3% of the STEM students) was in good mental health (as defined above) at baseline (p = 0.41).

The correlation between general and mental health was weak, but statistically significant (Spearman’s rho 0.19, p < 0.01).

### Predictors of general and mental health

For the general health of medical students, logistic regression revealed that a higher level of satisfaction with life, as well as a lower level of career ambition and physical activity of 1 hour or more per week were statistically significant predictors. Age was also a significant predictor for general health, whereas gender and mental health had no statistically significant effect (Table 4).

**Table 4:** Predictors of general health – medical students

**Predictor**	**Range**	**Odds ratio**	**95% CI**	**p-value**
Age	17-41	1.12	1.01-1.28	0.03
Gender	0 male	0.78	0.33-1.84	0.57
	1 female			
Mental health	0 no distress	1.13	0.36-3.56	0.83
	1 distress			
Satisfaction with life	0-16	0.75	0.64-0.87	<0.01
Career ambition	0-16	1.33	1.13-1.56	<0.01
Physical activity	0 less than one hour per week	0.34	0.14-0.79	0.012
	1 one hour or more per week			

Nagelkerkes R^2^ 0.24.

For the general health of STEM students, logistic regression revealed that a lower level of neuroticism and a higher level of satisfaction with life were statistically significant predictors. Age, gender and mental health had no statistically significant effect, with a similar odds ratio (OR; 1.10) for every one year increase in age compared to medical students (OR 1.12; Table 5).

**Table 5:** Predictors of general health – STEM students

**Predictor**	**Range**	**Odds ratio**	**95% CI**	**p-value**
Age	16-36	1.10	0.98-1.23	0.11
Gender	0 male	1.02	0.62-1.67	0.95
	1 female			
Mental health	0 no distress	1.72	0.88-3.35	0.11
	1 distress			
Satisfaction with life	0-16	0.88	0.79-0.97	0.01
Neuroticism	0-60	1.816	1.13-2.93	0.02

Nagelkerkes R^2^ 0.14.

For mental health of medical students, age was a statistically significant predictor. Here, lower levels of neuroticism and conscientiousness and higher levels of emotional distancing and, notably, striving for perfection and the subjective significance of work, showed a statistically significant contribution to predict good mental health. Gender and mental health had no statistically significant effect (Table 6).

**Table 6:** Predictors of mental health – medical students

**Predictor**	**Range**	**Odds ratio**	**95% CI**	**p-value**
Age	17-41	1.15	1.02-1.30	0.02
Gender	0 male	2.80	0.97-8.12	0.06
	1 female			
General health	0	1.33	0.32-5.59	0.70
	1 good/very good			
Subjective significance of work	0.16	0.83	0.69-1.00	0.05
Striving for perfection	0-16	0.75	0.60-0.94	0.01
Emotional distancing	0-16	0.56	0.42-0.74	<0.01
Neuroticism	0-60	12.35	4.93-30.92	<0.01
Conscientiousness	0-60	3.32	1.05-10.52	0.04

Nagelkerkes R^2^ 0.50.

For the mental health of STEM students, neither age nor gender were statistically significant predictors. However, the OR for every one year increse in age (1.10) was again similar compared to medical students (OR 1.15). Lower levels of neuroticism and resignation tendencies and being generally healthy were signicifantly related to good mental health in STEM students (Table 7).

**Table 7:** Predictors of mental health – STEM students

**Predictor**	**Range**	**Odds ratio**	**95% CI**	**p-value**
Age	16-36	1.10	0.95-1.27	0.22
Gender	0 male	1.01	0.53-1.94	0.97
	1 female			
General health	0	0.40	0.20-0.80	0.01
	1 good/very good			
Resignation tendencies	0-16	1.27	1.13-1.43	<0.01
Neuroticism	0-60	5.56	2.91-10.7	<0.01

Nagelkerkes R^2^ 0.40.

## Discussion

### General and mental health

In the baseline survey of our longitudinal study on medical students’ health and its predictors, we found that most medical students were in good general and mental health. Their fellow students from other, mainly science and technology subjects, were in similar good mental health but less often in good general health. In both groups, students with higher levels of satisfaction with life were more likely to be in good general health. Those medical and STEM students with lower levels of neuroticism were more likely to be in good mental health.

The proportion of individuals rating their general health as good in our cohort of medical students is comparable to that in the similar age group (18-29) of a recent German general population sample [44]. In this sample, 89.1% of the female and 92.3% of the male respondents rated their health as good or very good. Medical students in our cohort matched these porportions quite well (90.7% of the male and 90.9% of the female students were in good health), whereas only 79.6% of the male and 79.9% of the female STEM students were in good general health.

The proportion of medical students free of clinically-relevant depression (96.6%) tends to be higher than proportions derived in other studies on medical students in their first years of medical school using the same cut-off (70.7% and 94.3%) [24,45] and the proportion of a similar age group (18-29) of a German general population sample (90.1%) [46]. This might be due to the very early timepoint of the baseline survey before the beginning of courses and, thus, before exposure to medical-school-associated stress. Another explanation might be that young people with clinically-relevant depression are less likely to qualify for and to start medical school. The proportion of medical students free of clinically-relevant anxiety in our cohort (89.7%) lies in between scores reported in comparable studies on medical students (79.7% and 93.2%) [23,45]. The high proportions of students with clinically relevant depression and anxiety scores in the study of Karaoglu et al. [45] might reflect cultural differences as it was conducted in Turkey.

Our results are also in accordance with the results Leahy et al. derived from a cross-sectional study comparing medical students’ distress levels with those of students of non-medical subjects [47]. In this study, students from non-health disciplines were significantly more distressed than students from health disciplines and 11% had overall been treated for a mental health problem, compared to 12% (medical students) and 14% (STEM students) showing clinically-relevant anxiety and/or depression (as defined above) in our study.

### Study-related behaviour and experience

When compared to the STEM students in our cohort and German early-year medical students surveyed in other studies [30,31], students in our cohort showed consistently higher sum-scores for the dimensions that form the domains of professional commitment and emotional well-being. With the exception of “offensive coping with problems”, they showed lower to equal sum-scores in the dimensions that form the domain of resistance towards stress. As neither the STEM students in our cohort nor the medical students in the other studies were surveyed before the beginning of any relevant courses (see “Strengths and limitations”), this difference might again be related to the timepoint of the baseline survey, indicating that resistance towards stress is a trait which can be considered as “improving” while being exposed to stress.

### Personality traits

Consistent with earlier results on personality traits in medical students [48,49], we found low scores for neuroticism and high scores for agreeableness. Jerant et al. [48] described the medical students in their sample as having higher levels of conscientiousness and agreeableness, similar levels of openness to experience and extraversion and lower levels of neuroticism, when compared to a general population sample. This is consistent with our results in comparison to a general population sample from German-speaking countries [50]. As Jerant et al. conclude, high levels of conscientiousness and agreeableness might be expected in medical students. The observed low neuroticism scores might also reflect some social desirability in the responses, as Jerant et al. propose.

### Predictors of general and mental health

Consistent with earlier research [5,51], lower neuroticism scores predicted better mental health in both groups of students. In a study by Tyssen et al. [52] of Norwegian medical students (n = 421), neuroticism was an independent predictor of perceived stress. Hochwälder [53] reports that low neuroticism scores were correlated with low scores of emotional exhaustion in nurses (n = 659).

With regard to the focus of our work on health promotion, it was important to see that regular physical activity was a significant predictor for general health, at least in medical students. The beneficial effects of physical activiy on health and well-being have been reported extensively [54,55]. Especially if follow-up results reveal a health-promoting effect of regular physical activity, leaving space in the curriculum for regular physical activity for students from the beginning of their courses onwards could be a promising, albeit difficult to enforce, health-promoting intervention [38].

Despite a growing body of literature that has also shown the positive effects of regular exercise on mental health [56,57], we were not able to show such an effect in our regression analyses. This is consistent with the findings of Buchmann et al. [58] who reported that depression and anxiety were not correlated to exercise in Freshmen medical students. Instead, we found that lower levels of resignation tendencies and higher levels of emotional distancing were correlated to being mentally healthy in medical students. The ability to distance oneself from work has been described as an important factor to avoid workaholism and burnout [59].

Surprisingly, a higher level of striving for perfection was also a predictor of being mentally healthy in medical students. This is in contrast to a number of studies that describe perfectionism as a major influence for medical students’ or physicians’ stress and burnout [60], and which interpret a relative decrease in the course of study as a sign of a healthy adaptation [30]. On the other hand, Enns et al. [36] discern between an adaptive and a maladaptive form of perfectionism and report that only the latter was associated with higher levels of depression in a sample of first to third year medical students. Likewise, perfectionism as imposed by others and not by oneself was found to cause negative mental health effects [61]. Fry [62] even described perfectionism together with optimism and humour as positive moderators of the impact of daily hassles on self-esteem and burnout in female executives. However, none of these studies examined the association between striving for perfection and mental health as early in the students’ course as ours did. It will therefore be of interest as to how perfectionism will develop and how it will predict mental health during the course of our study.

### Strengths and limitations

The major strength of this study is its longitudinal design and the high number of participants, this latter due to a high response rate, especially among medical students. We will be following a cohort of medical students (two consecutive classes) throughout their entire study period.

The generalisability of our results may be limited by the single-centred nature of the study. However, apart from special study courses at a few medical schools, medical education in Germany follows a very similar curriculum nationwide. The age and gender distribution of our sample resembles the nationwide distribution [63]. Our findings may thus be generalised beyond Lübeck University Medical School at a national level.

The annual presentation of results to the cohort may have an influence on the subsequent surveys, but we consider them an indispensable measure to keep participants motivated to respond to the yearly surveys.

One limitation of our study is the lower than expected initial response rate among the STEM students. Due to the fact that we conducted the baseline survey in the pre-course week, which mainly consists of Mathematics lectures, we may have missed those students feeling well enough prepared in Mathematics to skip this course. Despite the fact that we conducted two subsequent baseline data collection waves in the first week of courses, this may be a potential source of bias, as these students could have estimated themselves healthier than those feeling the need to take this course in order to pass the exams. Also, a higher share of female STEM students participated in our study, when compared to the whole student population. As gender did not predict any of the health outcomes in STEM students, the risk of bias resulting from this is probably low.

The response rate of over 90% among the medical students makes any selection bias very unlikely. In contrast to the STEM counterpart, the medical students’ pre-course week doesn’t contain any subject matter relevant for examinations, which may have led to a different answering behaviour.

### Implications for research and practice

The high percentages of medical students in good self-rated general and/or mental health at the beginning of their undergraduate education and the knowledge from earlier research, indicate that the health of these students is likely to decline throughout medical school, highlighting the urgent need for health-promoting and preventive interventions at a very early point in time. The resilience of students should be fostered by, for example, teaching stress-reduction skills and promoting social and personal well-being activities. Compared to empirical studies describing medical school stress and related symptoms, there are only a few articles describing health-promoting interventions, and only a small proportion of these have been evaluated using scientifically-rigorous methods [64]. Thus, further high quality evaluation studies are urgently needed. Since we found a particularly low prevalence of regular practice of a relaxation technique among the study participants at baseline, and because there is evidence that learning and practising a relaxation technique might have a positive influence on health outcomes [64,65], this might be a potential starting point for a health-promoting intervention.

Also, the finding of the relatively bad health of STEM subject Freshmen should be further investigated in larger, multi-center trials. This group of students could require special interventions to promote their health and well-being and keep the rate of premature drop-outs low.

## Conclusions

Here we describe the rationale for our longitudinal study in medical and STEM students and report the baseline characteristics of a cohort of 881 students. At baseline, medical students rated their general health better, but there was no significant difference in mental health compared to STEM students. Medical students showed higher scores for the personality traits of extraversion, openness to experiences, agreeableness and conscientiousness. Regular physical activity, a good ability to distance themselves from work, low resignation tendencies and low levels of neuroticism were predictors for general and mental health. Follow-up surveys will reveal the development of general and mental health during medical school and factors predicting the maintainance of a good health status. The final results of our longitudinal study will help answer the question of what keeps future doctors healthy.

## Abbreviations

AVEM: Work-related behaviour and experience patterns (German: Arbeitsbezogene Verhaltens- und Erlebensmuster); CI: Confidence interval; HADS: Hospital anxiety and depression scale; STEM: Science, technology, engineering, and mathematics; NEO FFI: NEO Five-factor inventory; NEO PI-R: NEO personality inventory - revised; OR: Odds ratio; SF-36: Short Form - 36; WHO: World Health Organization.

## Competing interests

The authors declare that they have no competing interests.

## Authors’ contributions

All authors were involved in the design of the study. TK conceived the study, participated in the collection of data, performed the statistical analysis and data interpretation and drafted the manuscript. YT participated in the collection of data, performed the statistical analysis and revised the manuscript for important intellectual content. MS critically revised the manuscript for important intellectual content and gave advice regarding statistical issues. EV participated in the collection of data, statistical analysis and data interpretation and critically revised the manuscript for important intellectual content. All authors read and approved the final manuscript.

## Pre-publication history

The pre-publication history for this paper can be accessed here:

http://www.biomedcentral.com/1472-6920/14/134/prepub

## Supplementary Material

Additional file 1 Domains and dimensions of study-related behaviour and experience and related sample questionnaire items. Click here for file
