# Supplementary material for: Health-promoting factors in medical students and students of science, technology, engineering, and mathematics: design and baseline results of a comparative longitudinal study
Source: BMC Med Educ. 2014 Jul 4;14:134. doi: 10.1186/1472-6920-14-134 (PMC4096732; doi:10.1186/1472-6920-14-134)
Supplement: Additional file 1 — Domains and dimensions of study-related behaviour and experience and related sample questionnaire items. [file 1472-6920-14-134-S1.docx]

## Additional file 1 – Domains and dimensions of study-related behaviour and experience and related sample questionnaire items.

| Domain | Dimension | Sample item |
| --- | --- | --- |
| Professional commitment | Subjective significance of work | Studying is the most important element in my life. |
|  | Career ambition | I want to achieve more in my career than most people I know. |
|  | Tendency to exert | If necessary, I will study until I am exhausted. |
|  | Striving for perfection | My work should never contain errors or deficiencies. |
| Resistance towards stress | Emotional distancing | Leisure time is leisure time – I don’t lose any sleep over studying. |
|  | Resignation tendencies | I find it difficult to cope with lack of success. |
|  | Offensive coping with problems | Lack of success doesn’t discourage me, but makes me try even harder next time. |
|  | Balance and mental stability | I don’t get upset easily. |
| Emotional well-being | Satisfaction with work | Up to this point in my career, I have experienced more success than disappointments. |
|  | Satisfaction with life | So far, I have been satisfied with my life. |
|  | Experience and social support | I have the full support of my family. |
